# The Alchemist, the Scientist, and the Robot: Exploring the Potential of Human‐AI Symbiosis in Self‐Driving Polymer Laboratories

**DOI:** 10.1002/marc.202500380

**Published:** 2025-07-16

**Authors:** Bahar Dadfar, Berna Alemdag, Gözde Kabay

**Affiliations:** ^1^ Karlsruhe Institute of Technology (KIT) Institute of Functional Interfaces (IFG) Department For Bioengineering and Biosystems Eggenstein‐Leopoldshafen Germany

**Keywords:** adaptive autonomy, artificial intelligence, human–AI collaboration, polymer informatics, symbiotic intelligence

## Abstract

Polymer chemistry research has progressed through three methodological eras: the alchemist's intuitive trial‐and‐error, the scientist's rule‐based design, and the robot's algorithm‐guided automation. While approaches combining combinatorial chemistry with statistical design of experiments offer a systematic approach to polymer discovery, they struggle with complex design spaces, avoid human biases, and scale up. In response, the discipline has adopted automation and artificial intelligence (AI), culminating in self‐driving laboratories (SDLs), integrating high‐throughput experimentation into closed‐loop, AI‐assisted design‐build‐test‐learn cycles, enabling the rapid exploration of chemical spaces. However, while SDLs address throughput and complexity challenges, they introduce new forms of the original problems: algorithmic biases replace human biases, data sparsity creates constraints on design space navigation, and black‐box AI models create transparency issues, complicating interpretation. These challenges emphasize a critical point: complete algorithmic autonomy is inadequate without human involvement. Human intuition, ethical judgment, and domain expertise are crucial for establishing research objectives, identifying anomalies, and ensuring adherence to ethical constraints. This perspective supports a hybrid model grounded in symbiotic autonomy, where adaptive collaboration between humans and AI enhances trust, creativity, and reproducibility. By incorporating human reasoning into adaptive AI‐assisted SDL workflows, next‐generation autonomous polymer discovery will be not only faster but also wiser.

## Graphical abstract

Can artificial intelligence fully replace humans in polymer discovery? How can we effectively harness AI's potential while preserving human creativity, ethics, and expertise? Does AI introduce new biases and interpretability challenges into SDL? This perspective aims to answer these important questions and advocates for symbiotic human‐AI intelligence in creating adaptive autonomous workflows for rapid, competent, ethical, and innovative polymer discovery.

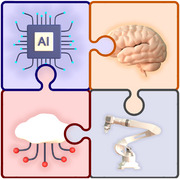

## 1 Introduction

Over the last century, polymer chemistry has evolved from an intuitive, trial‐and‐error approach to algorithmically driven, autonomous workflows. In 1920, Hermann Staudinger postulated that polymers consist of units of molecules linked together by the same covalent bonds found in smaller organic molecules. Following a decade‐long controversy, Staudinger's theory, which positions polymers as macromolecules rather than colloidal aggregates, was supported by experimental evidence and established the theoretical groundwork for macromolecular science [1].

Early breakthroughs often arise from human intuition, creativity, and sometimes serendipity. For example, polytetrafluoroethylene (PTFE, also known as Teflon) was discovered by accident in 1938 when Plunkett was experimenting with tetrafluoroethylene as a coolant for refrigerators and found a waxy solid clogging a gas line cylinder [2]. During the mid‐20th‐century polymer boom, successes were driven by heuristics: experts adopted a “cook‐and‐look” method, iteratively modifying recipes until they yielded transformative materials such as plastics, rubbers, and fibers that transformed society. However, this human‐centered strategy had significant limitations in terms of scope and speed. The range of possible monomers, polymer configurations, and processing conditions is extensive; relying only on intuition and hands‐on experimentation means that many potentially valuable polymers likely remain undiscovered.

By the late 20th century, scientists began adopting more systematic strategies to navigate chemical design space. Statistical design of experiments (DoE) [3, 4, 5] (e.g., factorial designs, response surface methodologies) and combinatorial chemistry [6, 7] (systematically generating and testing extensive libraries of chemical compounds under specified conditions) enable the structured exploration of the formulation variables and structure–property relationship (SPR) [7, 8]. While initially powerful, applying DoE‐based statistical approaches in conjunction with manual experimentation faced scalability, throughput, and adaptability issues as the number of tunable variables (e.g., monomer composition, initiator type, solvent environment, and reaction temperature) and performance objectives (e.g., yield, flexibility, and thermal stability) increased. As a result, these rule‐based methodologies became less practical.

This bottleneck motivated the adoption of high‐throughput experimentation (HTE) platforms in the 90s, which offered automation, miniaturization, and online analytics. HTE allowed researchers to test hundreds of polymer compositions in parallel, significantly accelerating SPR mapping. Pioneered by Symyx Technologies, robotics and automated analysis have been integrated into polymer screening workflows by Borealis AG [9] and several academic laboratories [10]. Running multiple reactions in parallel enabled a more comprehensive SPR evaluation, thereby accelerating the discovery of various original polymers.

Over the last decade, a convergence of advances in HTE and data science has given rise to AI‐assisted autonomous, or in other words, “self‐driving” laboratories, capable of designing, executing, and learning from experiments with minimal human involvement [11, 12]. While these autonomous technologies have already enabled the discovery of novel polymer families with properties surpassing known materials, significant challenges remain. These include data sparsity, biased or overfitting of the model, limited transferability across chemical spaces, and difficulty interpreting SPR with solely AI decision‐making. These issues suggest that algorithmic autonomy without human guidance is insufficient for advancing next‐generation polymer science [11, 13].

This perspective presents the methodological evolution of polymer chemistry, from early heuristic and rule‐based strategies to modern data‐driven discovery. We thoroughly analyze the inherent strengths and weaknesses of each approach, highlighting how AI and machine learning (ML) empower SDLs to independently evaluate experimental data and improve polymer synthesis within closed‐loop systems. It is also argued that true innovation in polymer science will not stem from full autonomy alone but from a symbiotic human–AI collaboration, where intuition, creative insights, and domain expertise are dynamically harmonized with algorithmic power to navigate and uncover the complexities of polymer design, with both learning from each other's strengths.

## 2 A Brief Historical Evaluation of Polymer Chemistry

### 2.1 Early Polymer Discoveries: From Myth to Method

The history of polymer chemistry is broad and multifaceted, starting with ancient practices guided by nature and intuition and evolving into modern experiments conducted by algorithmically guided robots. Ancient examples date back to 1600 B.C., when ancient Mesoamericans extracted latex from *Castilla elastica*, the rubber tree, then processed it with liquid from *Ipomoea alba* (a morning glory vine species) to manufacture rubber balls, hollow figurines, and other rubber artifacts [14]. In ancient Egypt, natural polymers were used in adhesives and embalming resins. Glues derived from animal collagen were widely used as adhesives, while tree resins such as myrrh and frankincense were used to formulate embalming balms for rituals. The Greeks and Romans utilized bitumen and pitch, hydrocarbon‐rich substances obtained from pine tar or petroleum seeps, for sealing and waterproofing ships and amphorae [15]. Although processed without modern chemical understanding, these materials represented early human engagement with natural polymers and formed the empirical basis for centuries of polymer research and discovery.

The shift from alchemical practices to empirical methods began during the Enlightenment. European reformers increasingly challenged dogma in favor of scientific inquiry, systematic experimentation, and the practical application of scientific knowledge. One notable figure in this transition is Charles Marie de la Condamine, who introduced “caoutchouc” samples, harvested from rubber trees (*Hevea brasiliensis*) to the European world in the 1730s. This prompted a wave of scientific investigations only years later by figures such as Joseph Priestley, who noted caoutchouc's ability to erase pencil marks and coined the term “rubber”, and Michael Faraday, in 1826, who explored its solubility features and analyzed its elemental composition [16]. Even though these early investigations were missing a theoretical foundation, they stimulated curiosity to understand materials better.

The 19th century witnessed significant breakthroughs, particularly in the development of polymeric materials. In the year 1839, Charles Goodyear made a significant discovery in the process of vulcanization by unintentionally subjecting rubber to heat in the presence of sulfur, which resulted in the formation of a material with enhanced durability and elasticity [17]. In 1861, British chemist Thomas Graham observed that certain organic compounds, such as cellulose, were dispersed in water; they did not crystallize and left sticky residues upon filtration. He hypothesized that these materials represented a different organization of matter, coining the term “colloids” from “kolla”, meaning glue. In 1869, Hyatt introduced celluloid, a thermoplastic derived from camphor and cellulose nitrate, a versatile plastic used widely in goods ranging from hair combs to silent film rolls [18]. Later, in 1907, Leo Baekeland synthesized Bakelite by combining phenol and formaldehyde under applied heat and pressure in a sealed autoclave. Bakelite was the first synthetic plastic, specifically a thermosetting resin, that demonstrated commercial success, particularly in the electrical industry, establishing the foundation of the “Age of Plastics” [19]. Though groundbreaking, these breakthroughs were mainly driven by heuristics, which means that empirical observations, chemical intuition, and accumulated experience guided scientists in selecting which monomers to combine, how to adjust reaction conditions, or which additives could influence the thermal or mechanical properties of the materials without a molecular‐level understanding of polymer structure and behavior.

A theoretical revolution in polymer chemistry began in the 1920s with Hermann Staudinger, who proposed that polymers were long chains of “covalently bonded macromolecules”, denying colloidal aggregate theories. Staudinger's foundational work marked the era of macromolecular science and earned him the Nobel Prize in 1953 [20, 21]. At the same period, Wallace Carothers applied this macromolecular view to synthesize nylon‐6,6 (1935) through the step‐growth polycondensation of hexamethylenediamine by adipic acid [22, 23]. This work was guided by the mathematical principles that later became the Carothers equation, linking monomer conversion and polymer chain length, guiding the optimization of the molecular weight in linear condensation polymers [24]. Carothers also pioneered the development of neoprene and early polyesters, but abandoned this work due to the low molecular weight of the resulting polymer [25]. In 1938, Paul Schlack synthesized Nylon 6, polycaprolactam, by ring‐opening polymerization of ε‐caprolactam. Though initially encountered by reproducibility issues, the process was refined in the 1960s with acyl‐lactam initiators.

### 2.2 Shifting Emphasis from Intuition to Theory

In the decades following World War II, the polymer industry experienced rapid growth driven by the increasing demand for innovative and cost‐effective materials. Advances in analytical characterization have led to the discovery of several new polymers and enhanced the understanding of the polymer structure–property relationship (SPR). Systematic experimentation, primarily grounded in empirical designs and early statistical methodologies, enabled numerous significant polymer discoveries, including polystyrene, poly(methyl methacrylate) (PMMA), polyvinyl chloride (PVC), polyethylene terephthalate (PET), polycarbonate (PC), and PTFE, and their industrialization which became foundational materials across multiple industries, from consumer products to advanced engineering applications [26].

As polymer chemistry matured theoretically, the field shifted from “cookbook” synthesis to systematic, rule‐based experimentation. Consequently, researchers moved beyond purely intuitive judgment, formalizing chemical knowledge into collections of “if‐then” rules or, in other words, rule‐based heuristic systematics, grounded in accumulated domain knowledge and supported by statistical reasoning [27, 28]. This hybrid strategy aimed to structure chemical exploration, improve reproducibility, and facilitate the systematic optimization of polymer properties. Karl Ziegler's discovery (1953) of high‐density polyethylene achieved through a combination of titanium tetrachloride and an alkyl aluminum co‐catalyst system enabled ethylene to polymerize under mild conditions [29]. The following year, Giulio Natta applied Ziegler's catalyst to propylene, synthesizing crystalline isotactic polypropylene. Their insight was that transition‐metal complexes could facilitate the formation of polymers [30]. Their insight into transition‐metal‐catalyzed polymerization enabled the controlled, mild‐condition synthesis of stereoregular polymers, earning them a Nobel Prize in 1963 [31].

Despite the rapid growth of polymer science, discovery remained slow and labor‐intensive, often struggling to meet industrial demands. Researchers mainly relied on one‐factor‐at‐a‐time experiments or small‐scale factorial designs (early forms of Design of Experiments, DoE) to fine‐tune polymer formulations. For instance, while optimizing Ziegler–Natta catalysts, metal halides and organoaluminium ratios were systematically varied in a pseudo‐grid search to enhance activity and stereoselectivity [30, 32, 33, 34]. While such methods were interpretable and grounded in chemical intuition, they were inherently limited by human bias and a lack of prior knowledge. Exploration was often confined to familiar monomers and reaction types, minimizing risk but frequently neglecting unconventional or counterintuitive chemistries. A representative example is the design of photoresist polymers, where multiple performance metrics (such as etch resistance, resolution, and sensitivity) must be optimized simultaneously. Even expert chemists tended to test only incremental variations of known styrene‐acrylate systems, following previous successful experimentations, and each design‐test‐analyze cycle could take weeks, further hindering the speed of innovation. Thus, heuristic methods achieved high success rates within narrow domains but lacked scalability. By the 1980s, the complexity of emerging polymers, from biomedical hydrogels to heat‐resistant thermoplastics, had outgrown what purely human‐guided discovery could handle.

One formalism that bridged intuition with a systematic exploration was the introduction of the design of experiments (DoE). Pioneered by Fisher [35] in the 1920s and later adapted to chemistry by Box and Wilson in 1951^4^, DoE provided a statistical framework for efficiently navigating multivariable systems. Unlike one‐factor‐at‐a‐time trials, factorial designs could capture interactions between variables with minimal runs. In polymer science, these statistical methodologies, including factorial designs, fractional factorials, and response surface methodologies (i.e., central composite design and Box–Behnken design) [36], have been widely adopted to optimize complex formulations, such as polymer blends, flame‐retardant additives, and catalyst systems [3]. DoE improved efficiency while enhancing interpretability, enabling researchers to identify key factor interactions (e.g., how initiator level and solvent polarity affect molecular weight)​. For instance, the synthesis of styrene‐butadiene rubber was streamlined in the late 1950s using a fractional factorial design, reducing hundreds of trials to 12 strategically selected experiments [5]. Rule‐based decision systems utilizing “if‐then” logic, such as SPR, also arose to encode known polymer property data into simple predictive rules. In parallel, factorial experimental designs (e.g., two‐level factorial arrays) enabled the systematic optimization of key reaction conditions, such as monomer ratios, temperature, and curing conditions, with minimal trial requirements.

While many early statistical approaches focused on SPR evaluation, others targeted improving polymerization yield, molecular weight distribution, and refining polymer performance. For example, Taguchi's orthogonal array design, introduced in the 1960s, further refined process robustness in polymerization by systematically reducing variability. Bicerano's book, “Prediction of Polymer Properties”, compiles extensive insights for estimating the SPR of polymers through their molecular structures, multiphase and interface modeling, and practical applications [37]. These early informatics tools were essentially human‐crafted rules generalized from data, a step beyond pure intuition, yet still far from data‐driven algorithms that characterize modern polymer informatics.

### 2.3 High Throughput Technologies: Accelerating Polymer Discovery through Automation

The growing demand for scalable, innovative materials with advanced features has shifted emphasis from trial‐and‐error methods toward high‐throughput experimentation (HTE). This shift led to the development of a family of high‐throughput technologies (HTTs) by adopting combinatorial chemistry and automated screening, which miniaturized reactions to microliter or even nanoliter scales and ran them in parallel. Yet, early HTT implementations remained human‐centered: experts chose variables based on their experience or literature, while automated equipment handled repetitive tasks, such as mixing in sealed parallel vessels and sampling, and offline analytics mapped SPR trends [7, 8]. For example, Kohn and coworkers pioneered a combinatorial HTE application in the polycondensation of 14 tyrosine‐based diphenols and 8 diacids, generating a 112‐member polyarylate library. This systematically developed library enabled them to elucidate how incremental structural changes influence polymer properties, such as hydrophobicity, glass transition temperature (*T_g_*), and even cellular response, outpacing that of sequential synthesis [7, 8].

Despite the demonstrated success of early HTEs, manual intermediate steps and offline characterization limited scalability. Around the same time, Symyx Technologies introduced a *“fully integrated high‐throughput workflow,”* initially demonstrated for the discovery of polyolefin catalysts. This platform utilized robotic liquid handlers to combinatorially mix metal precursors and ligands, followed by rapid screening assays to assess catalytic performance. End‐to‐end automation enabled the testing of dozens of catalyst candidates in the time it once took to run a single experiment, leading to the discovery of entirely new classes of olefin polymerization catalysts [38]. Polyolefin producers and academic laboratories soon adapted the same philosophy to polymer exploration, discovering chemistries that would have been impractical to probe manually.

As instrumentation matured, HTTs began to resemble miniature SDLs. Reactions were performed in multi‐well plates or microfluidic chips, utilizing massive parallelization. Within a single automated run, discrete factors, such as catalysts and ligands, alongside continuous parameters, including temperature, residence time, and pressure, can be varied, yielding detailed readouts of monomer conversion, by‐product profiles, solvent effects, and reproducibility online [39]. Modern HTE workflows increasingly embed statistical DoE into automation scripts, to ensure a balanced, information‐rich set of experiments to be performed without unnecessary repetition, so that every additional experiment maximizes what could be gathered about the structure–property landscape. Without a systematic design plan, running hundreds of reactions would otherwise lead to redundant data of limited value.

In 2004, Schubert's group linked a Chemspeed (ASW 2000) robotic synthesizer with inline size exclusion chromatography (SEC) and automated matrix‐assisted laser desorption/ionization time‐of‐flight (MALDI‐ToF) mass spectrometry [40, 41]. This setup, housed in an “inert” glovebox environment, automatically performed three polymerization reactions: ATRP, cationic ring‐opening polymerization (CROP), and emulsion polymerization, with immediate feedback on molecular‐weight distribution [40, 41]. The products of fully automated runs matched the performance of manual experiments and reproduced SPR trends (e.g., initiator‐dependent kinetics in CROP), at an accelerated pace. By the early 2010s, commercial platforms like Chemspeed's Accelerator and Swing could perform 16 or more parallel polymerizations, dispensing reagents with multi‐head robots and injecting samples directly into GPC or HPLC for inline analytics [42]. This integration effectively merged synthesis and characterization workflows into a single closed‐loop process, boosting discovery efficiency. Despite these advances, four main limitations remained: i) Fragmented workflow integration in many labs still separates synthesis, purification, and analysis steps, hindering overall throughput [43]; ii) the absence of unified, FAIR (Findable, Accessible, Interoperable, and Reusable) data repositories led to data silos and limited cross‐lab data comparisons [44]; iii) miniaturized reactions in multi‐well plates, impaired reproducibility mainly due to uneven mixing and heat transfer, affected data quality and cross‐platform consistency [45]; iv) scaling up from micro‐/nano‐scale to industrial volumes was not always straightforward, as reaction kinetics and surface effects differ at larger scales [46]. Even with these caveats, HTTs fundamentally changed the culture of polymer discovery. Rather than relying on iterative “cook and look” approaches, researchers adopted data‐rich exploration, which laid the physical and digital foundations for today's data‐driven SDLs.

## 3 The Data‐Driven Revolution: Polymer Informatics and Algorithm‐Guided Discovery

As computational power, automation capabilities, and data availability grew in the 2010s, polymer chemistry entered the era of polymer informatics: a field that utilizes ML and big‐data analytics for polymer discovery. Polymers with their inherent combinatorial complexity (ranging from various monomer sequences and molecular weights to sophisticated architectures), and ML excel at detecting patterns in such high‐dimensional data. Unlike heuristic “if‐then” rules or simple linear quantitative models (e.g., a linear regression model predicting boiling point from molecular weight and polarity), modern ML models can capture nonlinear and high‐dimensional relationships hidden in data, where multiple features jointly determine a property [10]. For instance, a polymer's *T_g_* depends on backbone flexibility, intermolecular forces, chain‐length distribution, and more features, typically too complex to decrypt by a single equation, yet one that ML can handle to uncover. The pattern recognition capabilities of ML, when implemented in HTTs, sped up the discovery of next‐generation polymers with properties surpassing the state of the art [10, 47, 48].

Crucial to this data‐driven progress was the creation of publicly available, curated polymer databases. A notable example is the PoLyInfo database of the Japanese National Institute for Materials Science (NIMS), which has compiled an extensive archive of manually curated chemical structures and descriptors from the literature [49]. Similarly, the Massachusetts Institute of Technology (MIT)’s Polymer Genome project provides a platform to encode polymer structures (via specialized SMILES strings) and utilize pre‐trained ML models to predict polymer properties [50]. With such informatics tools and open databases, polymer scientists gained access to vast amounts of data, enabling high‐performing AI/ML models that no single lab's data could have ever produced. As an example, Tao and coworkers benchmarked various ML algorithms (e.g., deep and convolutional neural networks, random forests, support vector machines) on a feature‐engineered dataset of approximately 7000 known polymers obtained from distinct polymer libraries. This process ultimately created a predictor capable of estimating the glass *T_g_* of approximately 5700 hypothetical homopolymers [51]. Similarly, Zhang and Xu used Gaussian process regression (GPR) to predict the *T_g_* of polyacrylamides utilizing quantum chemical descriptors derived from molecular structures. Even with only 37 training samples, they successfully captured SPR trends, demonstrating the predictive power of GPR despite the limited data [52]. Similar ML‐driven efforts have tackled polymer crystallinity [53], mechanical properties like elastic moduli (e.g., Young's modulus [54]), and optical traits like refractive index [55], demonstrating broad applicability.

Beyond forward property prediction and SPR evaluation, data‐driven approaches have enabled the inverse design of new polymers and other materials [56]. Generative models such as variational autoencoders (VAEs), generative adversarial networks can learn vast chemical design spaces and propose novel molecular structures that meet desired performance targets. For example, Zheng and coworkers combine a graph‐based VAE with high‐throughput molecular dynamics (MD) simulations to design vitrimers (recyclable polymers) [57]. Their model generated approximately 1 million hypothetical vitrimer chemistries and estimated *T_g_* for 8424 candidates and proposed new candidates with target *T_g_* values *(T_g_* ≈ 323 K), even beyond the training data range. One such vitrimer was found to have *a T_g_* of approximately 315 K, along with desirable self‐healing and flow properties, demonstrating how data‐driven insights guided by expert knowledge can accelerate the discovery of sustainable polymers.

While inverse design enables the discovery of potential polymer candidates, another significant advantage of ML is the efficient optimization of experimental design spaces for predefined performance goals (e.g., maximizing *T_g_* or yield). One such approach is Bayesian optimization (BO), a probabilistic and sample‐efficient strategy for optimizing expensive or complex functions [58]. The BO typically works by building a surrogate model, often a Gaussian process, to predict the relationship between inputs (e.g., temperature, catalyst type) and the target properties (e.g., yield, molecular weight). An acquisition function then balances exploration versus exploitation by suggesting which experiment to run next, updating the surrogate as new data are fed. This iterative process can identify optimal reaction conditions in far fewer trials than brute‐force or grid searches [58]. BO has been successfully applied to various experimental design optimizations, ranging from tuning stereoselective catalysts for ring‐opening polymerization [59], to improving the copolymerization of high‐density polyethylene [60], and to multi‐objective optimization of additive manufacturing resin recipes [61].

For example, a recent study employed a single‐objective BO application to minimize compositional drift in the free radical copolymerization of styrene and methyl methacrylate in a continuous‐flow reactor [62]. The challenge was that differing monomer reactivities cause the copolymer composition to drift from the feed ratio. By employing BO with two different batch sizes per iteration (a “sparse” 4‐candidate vs. an “exhaustive” 40‐candidate strategy), the authors quickly converged on conditions that held the copolymer composition near the target. The sparse BO found an optimal formulation in just five iterations, while the exhaustive BO revealed there were multiple viable solutions (identifying solvent: monomer ratio as a crucial variable). This demonstrated BO's efficiency, validating that even with limited runs, BO could mitigate compositional drift. This study makes three key contributions: 1) validation of BO as an effective tool for minimizing compositional drift with limited experimental runs, 2) quantification of solvent‐mediated kinetics in the copolymerization process, and 3) establishment of the infrastructure for multi‐objective BO, aiming to optimize copolymer composition and physical properties simultaneously. These findings suggest that BO can be extended to other polymerization systems requiring precise microstructure control to tailor material properties.

While traditional BO is typically applied to optimize a single objective, such as maximizing yields or minimizing dispersity, many real‐world polymer systems involve multiple, often competing objectives, including achieving high molecular weight while maintaining low dispersity. To address this complexity, multi‐objective BO (MOBO) balances trade‐offs among multiple performance metrics by identifying Pareto‐optimal solutions. This approach enables the simultaneous and data‐efficient optimization of several objectives within a unified modeling framework, making it particularly valuable for guiding complex materials design tasks [58]. For example, a MOBO approach is applied to optimize the production of polyvinyl acetate (PVAc), aiming to achieve a balance between product quality and manufacturing cost [63]. Recognizing the intrinsic trade‐offs in synthesizing long‐chain branched polymers, the authors applied MOBO with a q‐Expected Hyper‐Volume Improvement (qEHVI) acquisition function to identify optimal reaction conditions. The approach drastically reduced computational demand by modeling the polymerization process with differential equations and leveraging the data efficiency of Bayesian inference. Such results demonstrate that MOBO can efficiently navigate trade‐offs in complex polymer syntheses, identifying optimal conditions with orders of magnitude fewer trials than traditional methods, thereby highlighting the significant impact of incorporating multi‐objective decision strategies on autonomous optimization.

## 4 Self‐Driving Labs: Toward Autonomous Polymer Discovery

An SDL is an extension of traditional HTE platforms, coupling robotic hardware, real‐time feedback, and AI‐based decision‐making. These closed‐loop platforms can autonomously design, execute, and analyze experiments without direct human intervention, iteratively improving their strategies with each cycle [11, 12]. Unlike standard automation, which operates static, predefined protocols, SDLs dynamically adapt their workflows based on real‐time feedback, enabling adaptive exploration of synthesis and processing conditions [64].

A typical SDL is structured around hardware, software, and a data infrastructure layer, similar to the body, brain, and memory of a biological system [64]. In this configuration, the hardware mimics the physical body, performing operations traditionally performed by hand, such as dispensing reagents and transferring samples between the synthesis and characterization modules. It includes robotic components, such as reactors, mixers, heaters, and flow systems, for synthesis; as well as analytical instruments, including spectrometers, chromatographs, and microscopes, for characterization [11]. The software component acts as the brain, coordinating all hardware actions through custom scripting and orchestrating complex experimental workflows [11, 65, 66]. Above this, advanced algorithms, such as BO, serve as the cognitive cortex. These algorithms interpret incoming data in real‐time and determine the next set of experimental conditions, such as optimizing conductivity or minimizing dispersity, in light of predetermined research objectives [64]. Ultimately, the data infrastructure serves as the memory backbone, supporting both real‐time data acquisition and storage [12, 64]. This infrastructure also stores historical knowledge, such as literature data or prior experimental results, as well as metadata, sensor readings, and contextual annotations, all stored in centralized databases, providing the SDL with information to be utilized during decision‐making by data‐driven models. Comprehensive metadata standards enable traceability, reproducibility, and long‐term sustainability [11, 67, 68]. Meanwhile, real‐time dashboards provide both AI/ML systems and human operators with an interactive view of ongoing processes and results. Together, these components establish a self‐reinforced “design‐build‐test‐learn” cycle. AI algorithms design candidate experiments, the robotic hardware builds and tests them, instrumentation provides data for evaluation, and AI/ML models retrieve data from databases, actively learns, and continuously refine their predictive models, suggesting subsequent iterations. This closed loop can iterate quickly and even continuously, enabling SDLs to efficiently explore large, high‐dimensional design spaces and find optimal solutions much faster than traditional methods [11].

**FIGURE 1:**
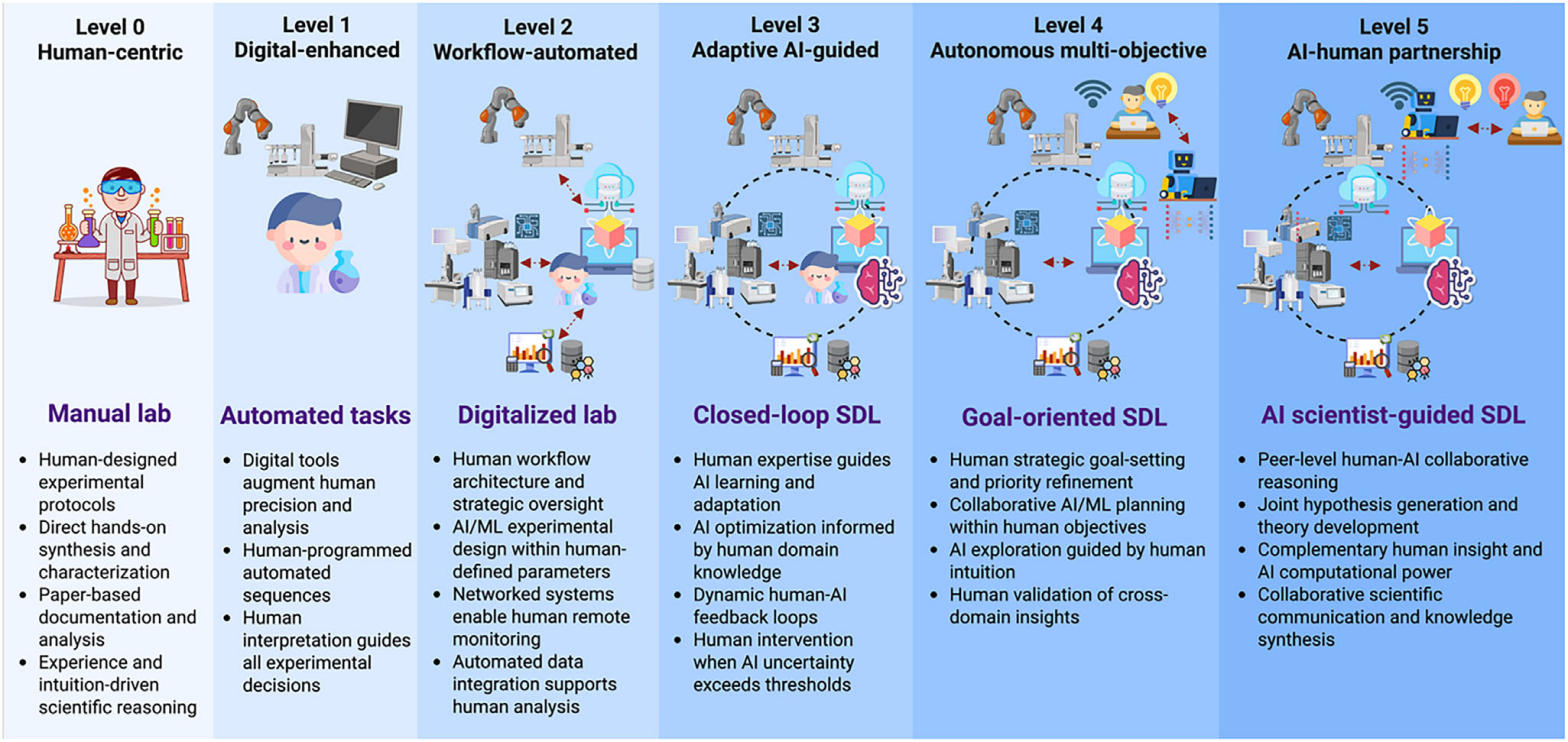
The evolution of human–AI collaboration in laboratory automation is explained through an illustrative polymer synthesis workflow. This framework presents six distinct modes of cooperation between humans and AI‐guided machines, which will be illustrated through a case study involving Dr. Mehmet Mutlu's work on biodegradable polymers in labs with varying autonomy levels. *Level 0* depicts traditional human‐centered scientific practice where Dr. Mutlu manually designs experimental protocols, weighs lactide monomers on balances, monitors reaction temperatures personally, and records all polymerization data in lab notebooks. His decisions depend on experience and direct observation of reaction progress. *Level 1* demonstrates digital enhancement of human abilities, where Dr. Mutlu continues to design protocols, while digital balances automatically log measurements, and temperature probes provide continuous data. He uses this improved data to make better adjustments to heating and reaction conditions. *Level 2* demonstrates workflow automation with strategic human oversight, where Dr. Mutlu programs robotic synthesis platforms to perform multi‐step polymerization sequences overnight. He designs automated protocols, reviews real‐time molecular weight data, and adjusts workflow parameters based on his analysis of reaction kinetics. *Level 3* illustrates adaptive AI‐guided collaboration, where Dr. Mutlu sets initial parameters and target properties, while AI systems monitor multiple reaction variables and make micro‐adjustments to optimize synthesis pathways. When the AI detects unexpected reaction intermediates, it flags anomalies for user interpretation, creating dynamic feedback loops between human knowledge and AI pattern recognition. *Level 4* features a goal‐oriented strategic partnership, where Dr. Mutlu defines overarching objectives for biodegradability, mechanical strength, and cost targets while collaborating with AI systems that design and run parallel experiments across multiple reactors. His research intuition guides resource decisions, while AI's computational power explores chemical space beyond his knowledge. *Level 5* exemplifies peer‐level partnership, where Dr. Mutlu and an AI scientist jointly propose new hypotheses combining insights from marine biology and synthetic chemistry. Together, they design experiments that neither could conceive alone, with AI modeling molecular interactions and Dr. Mutlu contributing to the understanding of synthesis feasibility and practical applications, leading to co‐developed research insights. The figure is created in BioRender under an academic subscription. (Kabay, G. (2025), https://BioRender.com/1gaa938).

As SDLs evolve, autonomy becomes a defining characteristic distinguishing them from conventional automated systems. With increasing sophistication, autonomy is no longer a binary concept but is instead classified across hardware and software axes [66]. These dimensions work in tandem; in other words, advances in hardware capabilities must be matched by corresponding software sophistication to progress between autonomy levels. Hardware autonomy refers to the degree of a system's control over physical tasks, ranging from simple actions, such as liquid dispensing, to complex ones, like inline sensing, sample transfers, and coordinating multiple instruments. Software autonomy refers to the AI's ability to plan, optimize, detect anomalies, and generate hypotheses. These two dimensions intersect to create discrete levels, where advancing hardware capabilities must be matched by corresponding software sophistication to achieve higher levels of autonomy. Combined, these axes yield a six‐level autonomy framework, from Level 0 (manual operation) to Level 5 (full autonomy). According to Tom and his team's analysis, most SDLs operate at Levels 2 and 3, where algorithmic decision‐making is combined with partial robotic task execution, with the long‐term vision of reaching Level 5 [66]. At Level 5, while systems operate with full hardware autonomy, they still require human oversight for strategic oversight and workflow co‐supervision. This persistent human involvement, even at the highest autonomy level, underscores the complex interplay between automated capabilities and human judgment, suggesting that advanced SDLs may be evolving toward sophisticated forms of human–AI partnership rather than pure autonomy. Figure 1 illustrates this six‐level autonomy within the context of polymer chemistry, demonstrating how each level does not eliminate human expertise but instead signifies its evolution and harmonization with increasingly advanced AI capabilities. Using the example of Dr. Mehmet Mutlu's development of biodegradable polymers for food packaging, the evolving workflow illustrates how human intelligence adapts and becomes more specialized, rather than diminishing, as technology advances. This progression suggests that increasing laboratory automation reflects a growing collaboration between humans and AI, rather than a shift toward sole software autonomy, with human intelligence adapting to provide more sophisticated cognitive input. At the same time, AI supports, rather than replaces, vital human expertise in polymer discovery.

This vision is already becoming a reality through pioneering SDL implementations. One such example is Argonne National Laboratory's “Polybot,” a modular platform that employed feature importance‐guided BO to navigate a 7D parameter space and identify optimal parameters to synthesize conductive polymer films with conductivities exceeding 4500 S cm⁻¹ [69]. Another example is Brabec's group's automated high‐throughput platform for synthesizing multicomponent polymer blend films for photovoltaics. In this design, the SDL autonomously varied the ratios of donor and acceptor polymers and solvent mixtures to maximize solar cell efficiency. By coupling BO with automated film deposition and screening, the system rapidly mapped a 4D composition space and identified blend ratios that yielded significantly improved power conversion efficiency and stability [70].

Cloud connectivity is another emerging feature used to construct data infrastructure in SDL, as it enhances modularity, remote access, and cross‐institutional collaboration. For example, Li and coworkers developed a remotely accessible microfluidic SDL for fully autonomous synthesis of perovskite nanocrystals [71]. The device combines robotic liquid handling with inline spectroscopy and a cloud‐integrated data management layer, allowing users to remotely launch, set goals, and monitor experiments in real‐time via a web interface. Knox and his team developed another SDL platform that integrates cloud‐based machine learning and orthogonal online analytics for the synthesis of polymeric nanoparticles [72]. They successfully demonstrated the multi‐objective optimization features of the ML algorithms, maximizing conversion and minimizing both molar mass dispersity and the particle polydispersity index, all while maintaining the particle size within a predetermined value.

These examples collectively demonstrate how AI‐assisted SDLs can handle various laborious tasks, from formulation to structural control, by navigating high‐dimensional parameter spaces. While these examples support workflows with little or no direct human intervention, the goal is not to eliminate scientists but to reposition them as collaborators in a human–AI symbiosis. In the following section, we explore this adaptive model, where human expertise complements algorithmic reasoning to create more robust, interpretable, and ethical autonomous SDLs.

### 4.1 Adaptive Autonomy: Symbiotic Human–AI Cooperation

As SDLs move toward increased autonomy, it is evident that full automation, while beneficial, is not always the optimal or most feasible choice, particularly in polymer discovery, where the chemical design spaces are vast and the available data can be limited or biased [73]. Rigid workflows may risk missing unexpected or creative acumens that often come from human intuition. To address this, hybrid autonomy models, which combine human intuition and machine intelligence, are evolving. These models combine AI's strengths in pattern recognition and computational efficiency with the scientist's ability to interpret context, apply domain‐specific knowledge, and think creatively. Studies in active learning have shown that “human‐in‐the‐loop” agents, where humans and machines collaborate to design and interpret experiments, outperform those performed by solely human or AI agents alone [74]. In this collaborative approach, AI excels at conducting in‐depth searches, running large‐scale experiments, and detecting correlations across data, while humans bring domain‐specific reasoning, anomaly detection, and hypothesis development. As a result, this synergistic strategy allows for more informed, adaptable, and innovative exploration of complex polymeric systems. This raises a critical question that one should ask: How can we determine when a machine should handle a task and when human intervention is essential?

Building on this hybrid strategy, the concept of adaptive autonomy arose to answer this critical question. Depending on the task context, adaptive autonomy dynamically allocates tasks between humans and machines in response to uncertainty, complexity, or the need for creativity [75]. For example, AI can autonomously execute routine SDL experiments, while human input is triggered when model uncertainty is high, a decision is risky, or expert opinion is required [76, 77, 78]. The ChemOS platform is a leading example of such adaptive autonomy integrated into SDL, enabling users to set experimental goals and parameters, then autonomously select reaction conditions and conduct experiments, pausing to seek human input when encountering unfamiliar situations or uncertain data [75]. Likewise, the Polybot system at Argonne National Laboratory links synthesis, processing, and characterization stations in a closed‐loop pipeline, while allowing for human intervention as needed [69]. The Autonomous Formulation Laboratory (AFL) extends this strategy to an open‐source and modular SDL architecture for complex soft matter formulations, combining ML with high‐throughput, reproducible data generation and real‐time feedback integration. The modular hardware–software architecture allows dynamic task allocation, demonstrating how human–AI teams can co‐pilot high‐throughput studies through adaptive autonomy [79]. By dynamically balancing machine automation and intelligence with human expertise and insights, these hybrid frameworks prevent blind spots, balancing reproducibility with creativity and safety [72].

### 4.2 Critical Aspects of Human–AI Collaboration

#### 4.2.1 Transparency: Interpretable AI Models and Visualization Tools

As ML models become integral to polymer research, transparency, interpretability, and explainability are critical for building trust in human–AI teams. Researchers must understand why an algorithm suggests a particular polymer or predicts a specific property, especially when results contradict conventional wisdom. Various explainability methods, such as inherently interpretable models [74, 80, 81, 82], symbolic regression [83, 84], post hoc [85, 86, 87, 88], attention mechanisms [89, 90, 91], and visualization tools (e.g., t‐SNE/UMAP embeddings or uncertainty maps) [51, 80, 92, 93], are being integrated into AI workflows to enhance transparency. Also, inherently interpretable “glass‐box” models can be used when applicable. For example, linear models and generalized additive models provide transparent relationships between inputs and outputs, allowing researchers to observe which molecular features define performance directly [74]. However, simplistic models, such as linear regression, shallow decision trees, or heuristic rule‐based systems, often fail to capture complex, nonlinear SPR [94]. Symbolic regression offers a more interpretable alternative that can uncover equations relating, for instance, the structural features of monomers to macroscopic polymer properties, providing clearer mechanistic insight than opaque black‐box models [74]. Researchers have even proposed using symbolic regression piecewise in different “regimes” of the data (identified via clustering) to maintain interpretability across a complex domain [74]. On the other hand, for high‐dimensional models that remain essentially black boxes (e.g., deep neural networks or graph neural networks), post hoc explanation techniques are increasingly employed to address interpretability issue by identifying the most influential features, verifying model reasoning against chemical knowledge, and guiding experimental design in polymer chemistry and materials science. Methods like SHAP and LIME can highlight which input features most influenced a specific prediction, albeit on a local case‐by‐case basis [74]. While not providing a complete mechanistic understanding, such techniques offer valuable insights and help to identify false correlations or domain‐insensitive predictions.

Visualization tools bridge the interpretability gap by translating abstract model representations, such as attention weights or latent embeddings, into intuitive formats that enable human researchers to act upon them. For instance, attention‐weight heatmaps in a transformer‐based polymer predictor, such as TransPolymer [93] can be visualized to show which parts of a polymer sequence the model “attends” to most for a given property prediction, implying that it has learned SPR [92, 93, 95]. Dimensionality reduction plots (e.g., UMAP or t‐SNE) of learned polymer embeddings can reveal clusters of chemically similar polymers in the model's latent space [96, 97, 98]. Likewise, uncertainty maps can be displayed to show specific regions of the design space where the model is uncertain, informing users where more data or caution is needed. Interactive dashboards are also handy, as users can adjust input parameters or constraints and immediately see the model's predictions or recommendations update, creating a two‐way dialogue between the AI assistant and the user [99, 100, 101]. Such visual analytics builds user trust and encourages researchers to reintroduce their intuition into the loop, enabling users to guide, validate, and correct AI behavior if necessary, critical attributes in hybrid workflows where human judgment and algorithmic prediction must co‐evolve.

For non‐experts or students, simple explanations can be generated in natural language to summarize why the model made a specific choice. Indeed, an exciting approach is rationale generation, which produces human‐like explanations for an AI's behavior [102, 103]. These rationales do not fully unveil the black box, but they translate the algorithm's decisions into narratives that a human can understand. Studies have demonstrated that human‐like justifications, even if approximate, greatly improve users’ trust and comprehension of autonomous systems. In one study, researchers trained a neural network to provide explanations, much like a human would justify a decision. While these explanations did not precisely reflect the AI's internal reasoning, they increased people's willingness to trust and collaborate with the AI [103]. This work highlights that explainability for non‐experts is not just a nice addition, but a requisite, especially in interdisciplinary fields like polymer science, where end‐users may not be AI experts. Multiple forms of interpretable feedback (e.g., visual, mathematical, linguistic) enable bidirectional knowledge transfer between humans and AI agents: the AI reveals SPRs, generates hypotheses, and optimizes design parameters, while humans gain insights into the AI's decision‐making process and grasp its limitations, guiding subsequent model refinement and prioritization [74].

#### 4.2.2 Explainability and Trust: Human–AI Co‐Design

For hybrid human–AI systems to reach their full potential, explainability and trust must be explicitly engineered into every stage of the system lifecycle. It is insufficient for the AI to be powerful; its decisions and suggestions must be transparent, interpretable, and justifiable to the human collaborators [74, 103]. This requirement is underlined in the human–machine co‐design concept, wherein humans assist in designing the AI's decision‐making processes, constraints, and objectives that align with human reasoning patterns and domain‐specific values.

Practically, co‐design means incorporating domain knowledge and human‐provided constraints into model architectures (for instance, encoding known chemical rules or safety limits prevents AI from proposing irrelevant or dangerous experiments). Additionally, it requires developing AI systems that can explain their rationale in user‐friendly terms. When humans and AI collaboratively design experiments and models, the workflow reduces trust issues. Humans are not left uncertain about the machine's motives, and the machine is less prone to exploring irrational areas due to human guidance. For example, an AI agent may propose a polymer synthesis that is theoretically optimal but uses a toxic reagent or an impractically long reaction time. If so, a human expert can promptly reject these options and recognize the limitations of AI for further design adjustments.

Over time, reinforcement learning or user feedback loops can enable the AI system to infer implicit preferences, evolving toward context‐aware optimization strategies that better reflect real‐world laboratory conventions [103]. It is also important to mention that this co‐design process is reciprocal, meaning that, similar to how AI systems learn from human constraints and corrections, humans must adapt by developing algorithmic literacy, refining their hypotheses based on machine‐generated insights, and becoming comfortable with probabilistic or counterintuitive outputs. Through collaborative goal‐setting and interpretive feedback, co‐design stimulates mutual calibration: the AI adjusts to what the human considers valuable or valid, while the human learns to identify new opportunities, patterns, or design pathways that the AI reveals. This mutual trust is a critical component of enduring and symbiotic human–AI collaboration in SDLs.

#### 4.2.3 Uncertainty Quantification and Active Learning

A cornerstone of human–AI collaboration in experimental design is quantifying uncertainty. Rather than viewing uncertainty as a weakness, hybrid workflows treat it as an informative signal for guiding exploration. Understanding what the model does not know is crucial for deciding when to explore new experiments or seek human input​ [72].

Modern ML models can quantify uncertainty in their predictions. For example, Bayesian neural networks yield a confidence interval with each property prediction, distinguishing the design space regions where the model is extrapolating with sparse or low‐quality data [76, 104, 105]. By identifying predictions with high epistemic uncertainty, which always stems from model limitations such as insufficient data or unfamiliar inputs, and high aleatoric uncertainty arising from inherent noise in the data, the system can flag those situations for closer inspection [72, 76, 77, 78]. In light of these predictions, an SDL might automatically suggest a new iteration in the region with the highest model uncertainty, assuming that new data in this region would reduce overall uncertainty. This forms the basis of active learning, where the AI proactively selects the most informative subsequent experiments to perform, instead of passively analyzing a predetermined dataset. Rather than relying on random or exhaustive searches, it directs experimental resources toward resolving uncertainties or marking a balance between exploration and exploitation in design space.

Active learning acts as a dynamic bridge between algorithmic exploration and human insights [106, 107, 108, 109, 110]. Instead of an exhaustive search, it prioritizes experimental resources toward resolving knowledge gaps. For instance, the Knox team recently demonstrated a cloud‐connected SDL that enhanced polymeric nanoparticle synthesis by combining active learning with real‐time analytics, effectively balancing various objectives such as particle size, shape, and yield [72]. Their system could autonomously navigate within the complex design space more efficiently than manual methods. Simultaneously, a remote team of scientists monitored and intervened as necessary via cloud interfaces, demonstrating successful utilization of adaptive autonomy.

To sum up, by quantifying models’ confidence level or uncertainty, AI systems know when to ask for help or defer decisions, functioning as a primitive machine “self‐awareness” that complements human judgment. The result creates a closed‐loop feedback cycle (a cyclical process in which outputs from a system are used as inputs for future iterations), wherein the model's uncertainty prompts new experiments, those experiments generate data, and the human expert interprets outcomes, adjusts hypotheses, or corrects the system. In this continuous cycle, AI gains confidence and precision, while the human researcher maintains epistemic control and scientific direction.

#### 4.2.4 Virtual Agents for Collaboration and Communication

One of the most game‐changing developments for human–AI workflows is the rise of large language models (LLMs), such as GPT‐4, and their domain‐specific counterparts (e.g., ChemCrow in chemistry). These models act as intelligent assistants or scientific mediators in the lab [111, 112]. Instead of requiring a human to write code or manually query databases, an LLM‐based system can understand a researcher's questions or instructions in their natural language and translate them into actionable steps. For instance, a chemist who wants to perform polymer synthesis might ask in plain English, “What monomer should I try next to increase the *T_g_*?”. The LLM can interpret this query, combine knowledge from literature and the lab's databases, integrate them into the lab's knowledge base, and suggest an experiment or retrieve relevant data, complete with an explanation. Models like GPT‐4 are already used to generate experimental procedures and optimize protocols from text inputs [111]. On top of that, LLMs enable conversational interfaces for complex AI systems: a researcher can interact with the model, refining questions or constraints in real time, much like collaborating with a virtual colleague. This vividly lowers the barrier for non‐experts to engage with AI‐driven platforms. Early applications, such as ChemCrow, have demonstrated that an LLM augmented with chemistry tools can assist in writing simulation code, controlling lab instruments via text commands, and even devising *de novo* molecular structures, all through a chat‐based interaction [112]. By serving as a bridge between human language and machine‐level execution, LLMs allow the unified integration of human expertise into autonomous workflows and vice versa. Humans can describe goals or constraints, and the AI interprets and acts while explaining its reasoning in understandable terms. This level of accessibility and transparency establishes trust, encouraging more people to utilize AI tools without requiring specialized training.

#### 4.2.5 Human–AI Synergy in SDL Practice

The polymer informatics field is rapidly evolving through the accumulation of examples, which are highlighting the potential of adaptive autonomy. On the AI side, polymer‐savvy models such as TransPolymer and polyBERT treat polymer sequences as a language, utilizing transformer neural networks to learn rich representations of polymer chemistry [92, 93, 95]. TransPolymer utilizes self‐attention and chemistry‐aware tokenization of polymer chains to predict properties with high accuracy, leveraging pretraining on large unlabeled polymer datasets (similar to how GPT‐4 is pre‐trained on text) to capture chemical context. Meanwhile, polyBERT employs multitask learning that maps these learned polymer fingerprints to multiple property predictions, enabling the simultaneous prediction of performance metrics. This machine‐learned fingerprint approach outperformed traditional, manually constructed descriptors by two orders of magnitude in speed while maintaining predictive accuracy [92]. Such models can be integrated into autonomous workflows to provide instant predictions of a candidate polymer's attributes, enabling researchers to screen possibilities or identify anomalies rapidly.

At the same time, advanced robotic platforms and high‐throughput synthesis pipelines are expanding experimental capabilities in SDLs. A potential adaptive human–AI workflow may, for example, use polyBERT's fast property predictions to narrow down a vast library of potential polymer structures to a manageable subset, employ an active‐learning‐driven experimentation to synthesize and test the top candidates, and finally rely on the human expertise to interpret results, assess failure modes and refine the design objectives. In a recent demonstration, Polybot autonomously performed hardware actions while an AI planner optimized a polymer formulation [69]. Yet, researchers remained “in the loop” to refine goals and enforce safety constraints, highlighting that neither humans nor machines alone can achieve the same speed and scientific insight as human–AI cooperation allows.

Notably, such hybrid workflows not only benefit from human input but also enable humans to learn from AI outputs. Models trained on vast chemical datasets can reveal unexpected patterns, suggest new candidates, or challenge common sense, stimulating new scientific questions, if taken seriously. This bidirectional flow of reasoning, which allows both humans and AI to learn from each other, forms the core of symbiotic autonomy‐guided SDL.

**FIGURE 2:**
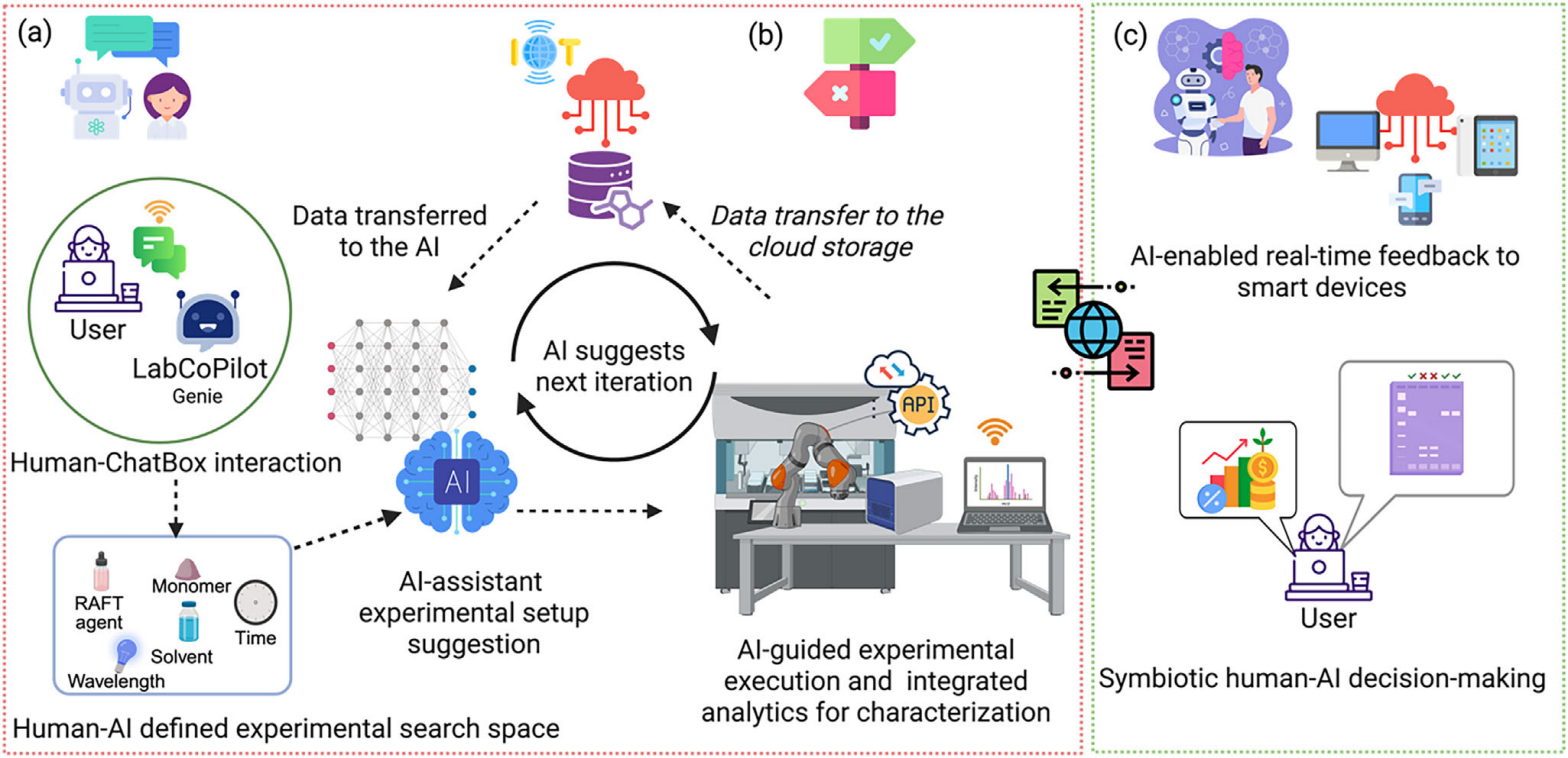
Conceptual schematic overview of an adaptive SDL workflow enhanced by symbiotic human–AI collaboration for sustainable polymer synthesis via PET‐RAFT polymerization. The user initiates the process by setting research goals and performance objectives through the ChatBox interface. LabCoPilot Genie searches chemical databases to determine the virtual design space, recommends HEMA, and proposes a set of reaction conditions for synthesis. Upon user confirmation, the robotic platform executes the experiments, while inline analytics continuously monitor key outputs such as monomer yield and molecular weight distribution. Experimental data are transferred to cloud storage, enabling real‐time remote data access and monitoring, multi‐user collaboration, and model retraining. As data accumulates, the AI learns and uncovers hidden SPR patterns using active learning strategies, suggesting subsequent iterations. Simultaneously, bidirectional feedback loops ensure continuous adaptation; the AI refines its suggestions based on evolving data and human feedback, while users can intervene to adjust goals, override AI decisions, interpret anomalies, or prioritize design trade‐offs (e.g., favoring biocompatibility over maximum yield). In parallel, AI‐generated insights are fed back to the cloud, connected smart devices, and user interfaces, facilitating remote monitoring, real‐time alerts, and informed decision‐making by users, as needed. This mutually adaptive architecture enables shared control, trust calibration, and the exchange of insights between human and AI agents. The platform emphasizes transparency, modularity, and openness through open‐source software and cloud connectivity, supporting decentralized, collaborative SDL operations across diverse research teams. The figure is created in BioRender under an academic subscription. (Kabay, G. (2025) https://BioRender.com/tp96c6w).

Figure 2 schematically represents a conceptual human–AI symbiotic workflow for photoinduced electron/energy transfer–reversible addition‐fragmentation chain transfer (PET‐RAFT) polymerization of sustainable biomedical polymers, using a hypothetical AI‐driven SDL assistant, “LabCoPilot Genie”. While individual components shown (such as robotic platforms, inline analytics, and cloud connectivity) are based on existing SDL technologies, the complete integrated system with conversational AI interface represents our proposed vision for next‐generation SDLs that operate with the highest level of hardware autonomy and human–AI symbiosis rather than describing any single existing implementation.

## 5 Summary and Outlook

In this perspective, we provided a historical overview of polymer chemistry, from early alchemical practices to systematic rule‐based methodologies and today's AI‐driven autonomous SDLs, revealing how the limitations of each period have catalyzed subsequent innovation. The emergence of polymer informatics and ML has addressed historical constraints through data‐driven pattern recognition and predictive modeling, with models such as polyBERT [92] and TransPolymer [93] demonstrating how AI can learn chemical language and predict properties with remarkable accuracy. SDLs exemplify symbiotic potential through adaptive autonomy frameworks, as shown by platforms like Polybot, which dynamically allocate tasks between human insight and algorithmic execution. Although there is an increasing demand to shift full software autonomy, we collectively believe that the actual breakthrough lies not in replacing human with AI, but in orchestrating partnerships where human creativity and AI computational power amplify each other's capabilities, transforming researchers into orchestrators of intelligent systems while maintaining essential roles in strategic oversight, ethical reasoning, and scientific interpretation.

Human–AI collaboration in self‐driving polymer laboratories is restricted by fundamental challenges that must be addressed to realize its transformative potential. The primary obstacle is the limitations of the current training datasets, which are often small, biased toward well‐explored materials, and lack the diversity needed for robust generalization. These constraints may create blind spots and lead to overfitting, ultimately limiting the generalizability, prediction performance, and restricting the real‐world applicability of these models [113]. Digital twins, virtual replicas of physical experimental systems, address this challenge by enabling safe, rapid, and cost‐effectively simulations to investigate “what‐if” scenarios before physical implementation. These virtual platforms integrate human intuition with AI predictions, providing interactive hypothesis testing while generating synthetic data to enhance training datasets [114, 115, 116, 117]. ML strategies, such as multi‐task learning (training models on multiple property prediction tasks simultaneously) and transfer learning (pre‐training a model on big data, then fine‐tuning it on specific tasks), further mitigate data‐related limitations by leveraging broad chemical knowledge to improve model robustness and promote cross‐task generalization [118]. These strategies also support community‐wide knowledge transfer, where one lab's HTE improves another lab's AI model. That knowledge can even help to predict a niche property with only a few experimental iterations [118]. The critical need is to establish more initiatives for data sharing, measurement standardization, and real‐time feedback from HTE systems to improve the dataset count and its relevance [119, 120, 121].

The persistent trade‐off between model complexity and interpretability presents both technical and cultural challenges. While explainable AI tools help demystify black‐box models, the inability of users to understand or question an AI's reasoning undermines their trust and adoption [122]. Integrating AI into SDL workflows requires a significant cultural shift; scientists must develop new skills to interpret uncertainty estimates, interact effectively with AI assistants, and critically assess machine‐generated hypotheses. In parallel, AI systems must be trained to accommodate human preferences, for instance, by allowing users to provide clear rationales for suggestions, justify outputs in understandable terms, and enable manual human overrides. One of the most effective ways to bring AI and humans closer together as colleagues is to enhance their communication. This can be achieved through implementing user‐centric interfaces and LLM‐powered chatbots in the SDL workflow, as these tools can facilitate natural language interactions, thereby enhancing transparency in AI decision‐making processes. Notably, special attention must be paid to fairness, bias, and the ethical use of AI to ensure diverse, transparent, and ethical recommendations, which are critical aspects of the successful implementation of human–AI symbiosis in SDL practice [103].

At the implementation level, human involvement in such AI‐driven systems provides critical benefits in SDLs. Expert oversight adds a trust layer in algorithmic decision‐making by validating AI‐generated hypotheses and ensuring accountability against the black‐box models [123]. By insisting on explainable, interpretable outputs, scientists connect complex model predictions with chemical intuition, allowing limited experimental datasets to be utilized more effectively [124]. Indeed, human experts excel at generalizing from sparse data, infusing first‐principles knowledge and symbolic reasoning so that AI systems can explain more observations with fewer experiments [125]. Equally important, human creativity and intuition inject innovative hypotheses and experimental directions that purely algorithmic searches might overlook [126]. The synergy of human insight and machine precision enhances the generalizability and reliability of SDL outcomes, enabling the identification and correction of model blind spots or biases while maintaining transparency, and ultimately building user trust in AI‐guided autonomous workflows [125].

Polymer synthesis operates according to well‐established physical and chemical principles (e.g., thermodynamics, reactivity, solubility) that purely data‐driven methods may overlook. Determining when to impose human domain knowledge remains critical. Explicit constraints, such as toxicity avoidance, can be easily encoded, whereas nuanced insights, like feasibility, prove more challenging to formalize. Hybrid models that combine mechanistic reasoning with machine intelligence have yet to evolve, while knowledge graphs and symbolic engines may bridge the gap between chemical validity and data‐driven creativity [118, 127, 128]. Despite these challenges, robotic platforms integrated with AI agents enable the synthesis and characterization of hundreds of polymer variants with unprecedented speed, creating opportunities for active learning approaches that focus resources on maximally informative experiments [94, 129, 130]. As costs fall and ease of use rises, more labs can deploy such material acceleration platforms, potentially networked via the cloud for global collaboration and simultaneously parallelized exploration.

Generative models represent a unique exploration capability by proposing novel polymer structures that extend beyond human intuition, as researchers evaluate their feasibility and accessibility [131, 132]. This co‐creative partnership has already yielded breakthrough discoveries in recyclable materials and biomaterials [133, 134], demonstrating how human–AI collaboration expands the known boundaries of polymer discovery. The most profound opportunity lies in advancing fundamental understanding of polymer science through data‐driven pattern recognition combined with human interpretation, enabling the discovery of new SPRs and theoretical principles that neither partner could achieve independently.

The next generation of SDLs will demand integration over replacement, with symbiotic intelligence reshaping how materials are discovered and knowledge is generated. This transformation redefines the human's role as an orchestrator of intelligent systems, setting research goals and performance targets, navigating algorithmic landscapes, and interpreting high‐dimensional data within mechanistic frameworks. Looking ahead, several emerging directions will define the horizon of symbiotic human–AI‐assisted SDL technologies for polymer discovery:

- As polymer databases grow in volume and variety, next‐generation models, such as polyBERT [92], TransPolymer [93], and graph neural networks, will continue to learn directly from chemical language and structure, capturing context‐aware, transferable knowledge that improves across tasks and domains.
- With LLMs like GPT‐4, domain‐specific co‐pilots such as ChemCrow, and dashboard‐driven workflows, humans will interact with AI systems in their natural language, supported by visualization or tactile simulation, making collaboration fluid and conversational, rather than code‐bound [131, 132].
- FAIR‐compliant datasets, model repositories, and shared benchmarks will become standard. As sequence databases revolutionized bioinformatics, structured polymer data and interpretable models will catalyze community‐driven discovery.
- Cloud‐based orchestration will further enhance fully integrated robotic platforms, such as Polybot or NIST's AFL, facilitating distributed and parallelized experimentation on a global scale.
- Decentralization is crucial for constructing durable and trustworthy networks of SDLs that securely share and verify experimental data, workflows, and discoveries across autonomous nodes, thereby eliminating the need for central authorities. Blockchain and smart contracts can support such infrastructure, ensuring data integrity, encouraging collaboration, and enabling the autonomous cooperation of tasks among distributed nodes [135, 136, 137].
- Future hybrid systems will not optimize solely for performance metrics. Instead, human‐in‐the‐loop constraints will incorporate environmental impact, safety, and equity as primary objectives, guiding polymer discovery toward responsible innovation.

The evolution toward symbiotic intelligence transcends the capabilities of imaginary Level 5 automation, as AI tools enhance scientific imagination and reveal previously hidden connections, while human scientists contribute their expertise, real‐world experience, and creativity. As the boundary between artificial and human intelligence blurs, polymer science will be characterized by adaptive autonomy and symbiotic intelligence: a blend of reasoning and data that is iterative, interpretable, and inclusive. This partnership will hence enable the exploration of previously inaccessible experimental territories, accelerating not only the speed of discovery but also the mechanistic understanding through rapidly tested and refined hypotheses. All in all, the upcoming age of polymer discovery, facilitating human–AI symbiotic partnerships in adaptive SDL workflows, promises to be not only faster but fundamentally wiser.

## Conflicts of Interest

The authors declare no conflicts of interest.
